# Radial microfibril arrangements in wood cell walls

**DOI:** 10.1007/s00425-022-03976-2

**Published:** 2022-09-10

**Authors:** Mona C. Maaß, Salimeh Saleh, Holger Militz, Cynthia A. Volkert

**Affiliations:** 1 Institute of Materials Physics, University of Göttingen, Friedrich-Hund-Platz 1, 37077 Göttingen, Germany; 2 Department of Wood Biology and Wood Products, University of Göttingen, Büsgenweg 4, 37073 Göttingen, Germany; 3 Present Address: Institute of Inorganic Chemisty, University of Göttingen, Tammannstrasse 4, 37077 Göttingen, Germany

**Keywords:** Cell wall layer wrinkling, Microfibril angle (MFA), Microfibril arrangement, S1/S2 interface, Ultrastructure, Whorl-like microfibril structure, Wood cell wall structure

## Abstract

**Main conclusion:**

TEM and AFM imaging reveal radial orientations and whorl-like arrangements of cellulose microfibrils near the S1/S2 interface. These are explained by wrinkling during lamellar cell growth.

**Abstract:**

In the most widely accepted model of the ultrastructure of wood cell walls, the cellulose microfibrils are arranged in helical patterns on concentric layers. However, this model is contradicted by a number of transmission electron microscopy (TEM) studies which reveal a radial component to the microfibril orientations in the cell wall. The idea of a radial component of the microfibril directions is not widely accepted, since it cannot easily be explained within the current understanding of lamellar cell growth. To help clarify the microfibril arrangements in wood cell walls, we have investigated various wood cell wall sections using both transmission electron microscopy and atomic force microscopy, and using various imaging and specimen preparation methods. Our investigations confirm that the microfibrils have a radial component near the interface between the S1 and S2 cell wall layers, and also reveal a whorl-like microfibril arrangement at the S1/S2 interface. These whorl-like structures are consistent with cell wall wrinkling during growth, allowing the radial microfibril component to be reconciled with the established models for lamellar cell growth.

**Supplementary Information:**

The online version contains supplementary material available at 10.1007/s00425-022-03976-2.

## Introduction

**Fig. 1:**
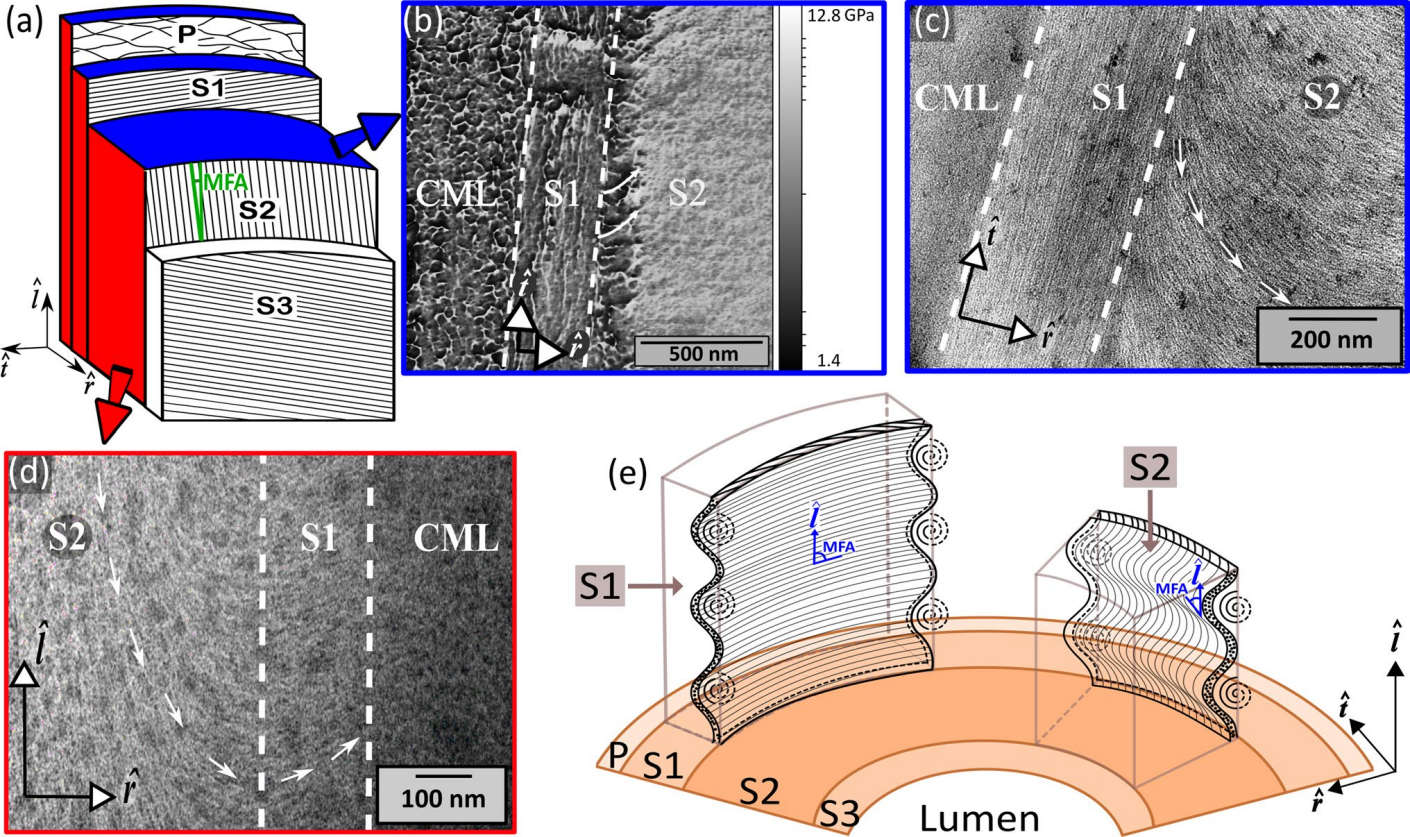
**a** Schematic picture of the cell wall structure with the microfibril angle (MFA) indicated in green. **b** AFM storage modulus map of the radial–tangential (r̂–t̂) section; white arrows are used to highlight the microfibril orientations. **c**, **d** TEM bright field images of an ultramicrotome prepared radial–tangential (r̂–l̂) section and a FIB prepared radial–longitudinal (r̂–l̂) section [reproduced from Maaß et al. (2020) under the terms of the Creative Commons CC-BY license.]. **e** Sketch of the envisioned 3D-microfibril arrangements in the wood cell wall derived from the 2D-section images

The excellent mechanical performance of wood is attributed to the complex hierarchical arrangement of the polymers from which it is made. Macroscopically, wood consists of annual rings made up of layers of thin-walled and thick-walled cells. These cylindrical, tube-shaped cells are connected to each other by a layer called the middle lamella. The cell walls themselves consist of a primary layer (P) and three secondary layers (Fig. 1a), each with its own composition and structure. The primary layer and middle lamella are often grouped together and called the compound middle lamella (CML). The outermost layer of the secondary wall is S1, a thin layer that makes up about 10% of the cell wall. The S2 layer is the central and thickest secondary wall layer, making up about two-thirds of the cell wall thickness. It makes the most significant contribution to the cell wall properties. The innermost relatively thin S3 layer is adjacent to the cell lumen. The layers are predominately made up of three polymers: cellulose, hemicellulose, and lignin, although the relative amounts vary from layer to layer. The cellulose is present as semi-crystalline elementary fibrils, which agglomerate to form microfibrils, and are embedded in the amorphous lignin matrix, while hemicellulose acts as a bonding agent between the microfibrils and the matrix. The arrangement of the microfibrils is different in each of the secondary wall layers, and is intensively discussed in the literature (Donaldson 2001; Fahlén and Salmén 2002; Zimmermann 2007; Reza et al. 2019). In the established model, microfibrils are arranged as helices within concentric layers of the cell wall (Ruel et al. 1978; Fahlén and Salmén 2002). The angle between the tangential component of the microfibril direction in the layers and the longitudinal axis of the tree or cell is called the microfibril angle (MFA) and is different in every layer (Fig. 1a): 60–80° in S1, 0–30° in S2, and 60–90° in S3. Reza et al. (2014) proposed that the microfibril direction also has a radial component in the cell wall, based on transmission electron microscope (TEM) studies. Although a radial component had already been observed in previously published TEM images (Hepler et al. 1970; Singh et al. 2002; Brändström et al. 2003; Fromm et al. 2003), Reza et al. (2014) appear to be the first to call it out. The idea of a radial microfibril angle (MFA) component is not widely accepted in the scientific community, because it is not easily reconciled with the current understanding of cell growth (Ruel et al. 2006; Salmén 2018). Cell wall growth occurs by layer-by-layer deposition of cellulose fibrils, starting from the outer primary layer and moving to the inner secondary layer, and cannot account for microfibrils that radially traverse the cell wall. On the other hand, the presence of a radial component has been recently further validated by real-time microscopic fracture studies, which show that cracks propagating along the radial direction account for the surprising splitting fracture toughness of wood (Maaß et al. 2020).

This paper presents a comprehensive study of the microfibril arrangements in late wood cell walls. In addition to transmission electron microscope (TEM) imaging, we use several imaging methods based on Atomic Force Microscopy (AFM), as well as several sample preparation methods, so that we can differentiate possible preparation and imaging artifacts from the actual wood structure (Toumpanaki et al. 2020). All methods and specimens confirm a radial component of the microfibril direction and reveal for the first time whorl-like microfibril structures at the S1/S2 interface. We argue that these arrangements are consistent with wrinkling of the cell wall layers due to growth stresses and can be reconciled with the existing models for lamellar cell growth.

## Materials and methods

*Pinus silvestris* L. and *Picea abies* (L.) H. Karst. were selected for this study, since they both are softwoods with a relatively simple cell structure mainly consisting of tracheid cells. To reconstruct the complex 3D structure of the microfibril arrangements in the wood cell walls, both radial–tangential (r̂–t̂) and radial–longitudinal (r̂–l̂) sections were prepared from wood that had been stored under ambient conditions. The r̂–t̂ and r̂–l̂ sections, which are described in terms of the cylindrical coordinate system of the tracheid cells, see Fig. 1a, were taken, respectively, from both tangential and radial walls and from tangential walls only.

The thin wood sections needed for TEM imaging (< 100 nm) were prepared using both ultramicrotomy and focused ion beam (FIB) machining. Ultramicrotome slicing was performed on epoxy embedded material using a diamond knife. The slicing directions were varied to distinguish any possible direction-dependent cutting artifacts from the actual cell wall structure. FIB sections were machined by sputtering with a focused 30 keV Ga^+^ ion beam. FIB irradiation damage and contamination, which show up as darkened regions in the TEM images, were kept to a minimum by working with low ion doses. No embedding material was used for the FIB specimen preparation, since there are reports that it may migrate into the cell walls (Coste et al. 2021), which might also result in rearrangements of the microfibrils. Further details of the ultramicrotome and FIB preparation methods are provided in the Supplementary Information.

The FIB and ultramicrotome prepared sections were investigated in a Philips CM12 TEM using a 120 kV electron beam. The specimens were first treated with a KMnO_4_ solution which specifically stains lignin, thereby providing the Z-contrast needed to detect the microfibril structure in the TEM (Hepler et al. 1970; Reza et al. 2015). It seems unlikely that the staining changes the structure. However, electron irradiation during TEM imaging does damage the samples; if the electron dose is too high, bubbles will form and grow inside the sample, presumably due to knock-on damage and volatile species formation. Altogether, 2 radial–tangential (r̂–t̂) and 14 radial–longitudinal (r̂–l̂) sections of the S1/S2 interface region were prepared and successfully imaged with the TEM.

Smooth sectioned surfaces were prepared for AFM imaging by microtome slicing of unembedded samples. Embedding material was avoided to maintain the contrast needed for topographical and nanomechanical AFM imaging. Although microtoming unembedded AFM specimens causes known characteristic artifacts due to knife defects and stick-slip motion during slicing (Casdorff et al. 2017), these can be identified by recording the slicing direction. AFM images of the microtomed radial–tangential (r̂–t̂) and radial–longitudinal (r̂–l̂) surfaces were recorded using bimodal AM–FM mapping (Rodríguez and García 2004; Proksch and Yablon 2012). The AM–FM method produces two resonances, the lower of which provides surface topography and phase lag, while the higher resonance provides the storage modulus and information about dissipation. Further details are provided in the Supplementary Information. Altogether, around 15 images from 5 radial–tangential (r̂–t̂) sections and around 10 images from 3 radial–longitudinal (r̂–l̂) sections of the S1/S2 interface region were successfully obtained with the AFM.

## Results

Representative images of the S1/S2 interface region were obtained using the different sample preparation and imaging methods and are shown in Fig. 1. In an AFM modulus image of the radial–tangential (r̂–t̂) plane (Fig. 1b), the isotropic globular layer is identified as the compound middle lamella, while preferred microfibril arrangements are detected in both the S1 and S2 layers. The S1 microfibrils have a concentric lamellar structure with no detectable radial component in the r̂–t̂ plane, while the region in the S2 near the S1 shows a clear radial component (highlighted with white arrows). We use the change in microfibril direction to define the position of the S1/S2 interface (Reza et al. 2019). Presumably, the change in contrast in the S2 layer roughly 100 nm from the S1/S2 interface is due to the increasing cellulose content while moving into the S2 layer (Panshin and de Zeeuw 1980; Brändström et al. 2003), since cellulose is significantly stiffer than lignin (Gibson 2012). The radial component near the S1/S2 interface is also visible in the AFM phase lag and dissipation maps (see Fig. S1 and S2 in the Supplementary Information). By varying the microtome cutting direction, it can be clearly distinguished from features caused by the knife which sometimes even obscure the microfibril structure (Fig. S3 in the Supplementary Information).

Bright field TEM images of FIB and ultramicrotome prepared sections also reveal the microfibrils in the S1 and S2 layers, which appear as white regions, because the lignin matrix is dark due to staining. As expected, microfibrils are not visible in the CML, since it nearly solely consists of lignin (Fig. 1c, d). TEM images of r̂–t̂ sections show the same microfibril arrangements as the AFM images, with a purely tangential arrangement in the S1 layer and both radial and tangential components in the S2 layer (Fig. 1c). In contrast, TEM images of r̂–l̂ sections (Fig. 1d) reveal a radial component of the microfibril arrangement in both the S1 and S2 layers, becoming purely radial at a distance of ca. 200 nm from the CML, in the presumed location of the S1/S2 interface. The FIB and ultramicrotome prepared specimens show the same microfibril arrangements, but the structure is more visible in the ultramicrotome than in the FIB prepared samples (see Fig. S4 in the Supplementary Information).

**Fig. 2:**
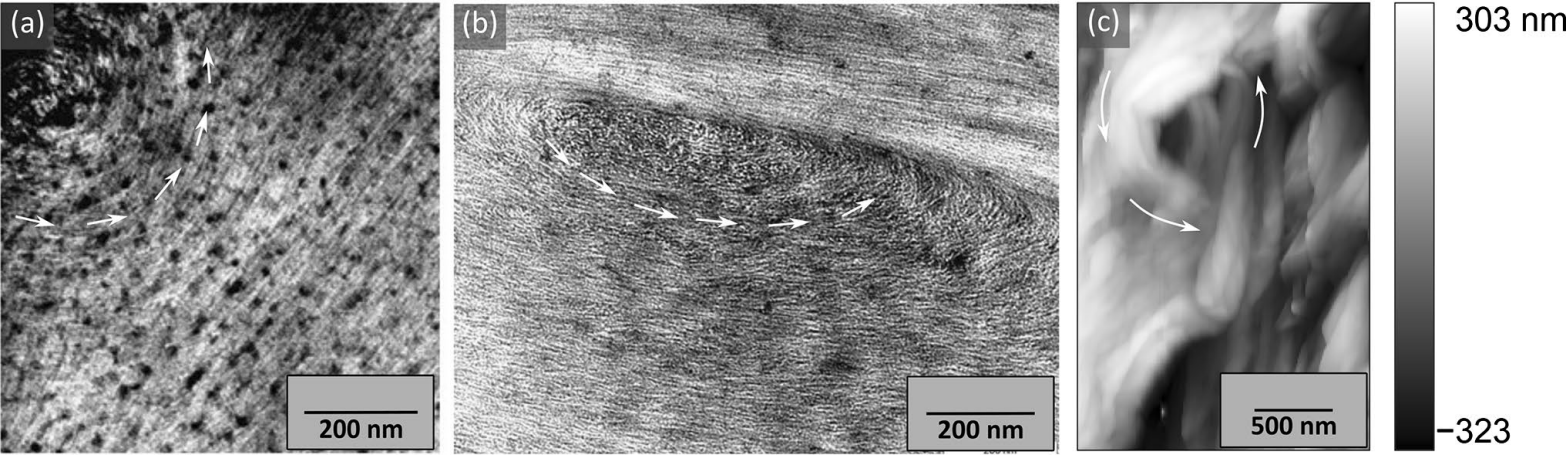
Images of whorl-like microfibril structures (microfibril directions indicated by white arrows) near the S1/S2 interface. **a**, **b** TEM images of a FIB prepared radial–longitudinal (r̂–l̂) section and an ultramicrotome prepared radial–tangential (r̂–t̂) section. **c** AFM height map of a radial–longitudinal (r̂–l̂) section. The dark spots in **a** are the result of excess KMnO_4_ stain

A number of specimens from the S1/S2 interface region of late wood cell walls were prepared and imaged, and showed similar microfibril arrangements to those shown in Fig. 1. However, sometimes whorl-like structures with a diameter of approximately 1 µm were observed along the S1/S2 interface (Fig. 2). They are present in both r̂–t̂ and r̂–l̂ sections, and could be resolved with all imaging and sample preparation methods.

## Discussion

The microfibril arrangements in 2D sections through the S1 and S2 layers of late wood have been investigated using different imaging and sample preparation methods. We find that the microfibril arrangements in the S1 layer have a strong radial component in the r̂–l̂ section (Fig. 1d) but none in the r̂–t̂ section (Fig. 1c), while the S2 layer near the S1/S2 interface has a strong radial component in both the r̂–t̂ and r̂–l̂ sections. The observed arrangements do not depend on the preparation or imaging method, making a strong case that they are indeed the true structure of the wood cell walls and not the result of artifacts of the sample preparation or imaging methods. In particular, the consistency of the images for the different specimen preparation steps indicates that FIB milling, epoxy embedding, and KMnO_4_ staining do not significantly change the microfibril arrangements. Also, ultramicrotome and microtome slicing do not appear to destroy microfibril arrangements, although features from the knife cutting process can be present and distinguished by varying the cutting direction (Fig. S1 in the Supplementary Information). By comparing AFM and TEM images, one can additionally conclude that electron beam damage also does not have a strong impact, as long as short exposure times and an appropriate accelerating voltage are used.

The most important conclusion from our study is that there is indeed a radial component to the orientation of the microfibrils (Fig. 1), sometimes even manifesting as whorl-like structures at the S1/S2 interface (Fig. 2). Radial–tangential (r̂–t̂) and radial–longitudinal (r̂–l̂) TEM images of the S1 and S2 layers in previous publications have also found a radial component in both soft and hard woods (Singh et al. 2002; Brändström et al. 2003; Fromm et al. 2003; Donaldson and Xu 2005), but it was only discussed by Reza et al. (2014). Here, we have reproduced and confirmed their results on pine and spruce wood using multiple imaging and preparation techniques. The consistency of our results with the literature results suggests that the radial component is a common feature of microfibril arrangements near the S1/S2 interface. In fact, a closer look at a study on pine wood (Donaldson and Xu 2005) shows that there is also a radial component at the S2/S3 interface. The existence of the radial component has remained largely ignored in the literature, presumably because it is not easily explained by widely accepted models for cell growth (Ruel et al. 2006; Salmén 2018). However, the strong evidence presented here and the fact that a radial component of the microfibril direction is necessary to explain the high splitting fracture toughness of wood (Maaß et al. 2020) motivates the need for discussion of possible ways to reconcile the observed structures and existing growth models.

Keeping in mind that the TEM and AFM image resolution is not always sufficient to detect individual microfibrils, so that the observed contrast also results from the layered structure of the microfibrils, we propose a 3D model for the microfibril structure that can account for all of the 2D-section images. Specifically, the concentric layers of helically arranged microfibrils must be wrinkled along the longitudinal cell direction (Fig. 1e). This leads to both the observed radial component of the microfibril directions and the whorl-like structures at the S1/S2 interface. Such an arrangement does not fundamentally deviate from the widely favored concentric layered helical structure of the microfibrils (Ruel et al. 1978; Fahlén and Salmén 2002), except we have introduced axial wrinkling to account for the observed radial components and whorl structures. Furthermore, the axial wrinkling can explain the high variability of the observed microfibril arrangements near the S1/S2 interface (Reza et al. 2019).

Wrinkling of microfibril layers has been previously discussed in the literature of cell wall growth models, motivated by shrinkage as a result of crystallization of the initially amorphous cellulose (Plomion et al. 2001). Since the cellulose content is higher in the S2 than in all other layers, the S2 will shrink more upon crystallization, creating stresses in the surrounding thinner layers (Lipowczan et al. 2018), which can lead to wrinkling, such as shown in Fig. 1e.

The longitudinal wrinkling and radial microfibril arrangements are expected to affect the mechanical properties of wood. It is already known that the radial component steers delamination cracks in the S2 layer toward the S1/S2 interface where the cracks can only propagate further by changing direction or fibril rupture, thus leading to an increase in toughness (Maaß et al. 2020). Furthermore, it seems likely that the locally varying radial component reduces the anisotropy in the elastic response of the cell walls as well as makes wood more robust against axial buckling instabilities. The full implications of the microfibril radial component on wood mechanical performance remain to be investigated.

## Conclusion

TEM and AFM images of the S1 and S2 layers of pine and spruce wood cell walls confirm the presence of radial components and reveal whorl-like arrangements of the microfibrils at the S1/S2 interface. Possible specimen preparation and imaging artifacts have been ruled out as the cause of these arrangements. A comparison with literature studies indicates that the radial component of the microfibrils is found in a variety of soft and hard woods, so we suggest that it is a common feature of wood cells. It can be explained by extending the cell wall structure model to allow for longitudinal wrinkling of the concentric microfibril layers near the S1/S2 interface, which has been postulated to result from stresses introduced in the cell wall layers during growth.

### *Author contribution statement*

SS and MCM: conducted experiments. HM: provided the specimens. All authors conceived the research, analyzed data, and wrote and approved the manuscript.

## Supplementary Information

Below is the link to the electronic supplementary material.

Supplementary file1 (DOCX 1637 KB)

## Data Availability

The data generated and analyzed during the current study are provided, in part, as supplementary material and are available from the corresponding author on reasonable request.

## Abbreviations

AFM: Atomic force microscope
CML: Compound middle lamella
FIB: Focused ion beam
TEM: Transmission electron microscope
