# Supplementary material for: Radial microfibril arrangements in wood cell walls
Source: Planta. 2022 Sep 10;256(4):75. doi: 10.1007/s00425-022-03976-2 (PMC9464115; doi:10.1007/s00425-022-03976-2)
Supplement: Supplementary file 1 — Supplementary file1 (DOCX 1637 KB) [file 425_2022_3976_MOESM1_ESM.docx]

**Supplementary Information**

**Radial microfibril arrangements in wood cell walls**

Mona C. Maaß^1^, Salimeh Saleh^1^, Holger Militz^2^, Cynthia A. Volkert^1^

The following sections provide more details on the experimental methods and support the discussion about the impact of known measurement artifacts on the results, using additional TEM and AFM images not shown in the article.

**Discussion of possible section preparation and imaging artifacts**

AFM imaging has been used in a number of studies to detect the structure of wood cell walls (Toumpanaki et al. 2020), and requires surfaces that are smooth on a length scale well below the length scale of the features of interest. The usual embedding methods that are used to prepare smooth surfaces by microtoming wood specimens should be avoided, as the embedding material changes the nanomechanical measurements. However, microtoming unembedded AFM specimens causes known characteristic artifacts due to the stick-slip effect of the knife and knife defects. For example, slick-slip lines are clearly visible as vertical lines in Fig. S1, but are easily distinguishable from the microfibril orientations indicated with white arrows. Moreover, different AFM imaging modes measuring different sample properties all show the same microfibril arrangement at the S1/S2 interface (Fig. S2). Thus, it can be ruled out that the radial component of the microfibril directions at this interface is an artifact of the sample preparation method.


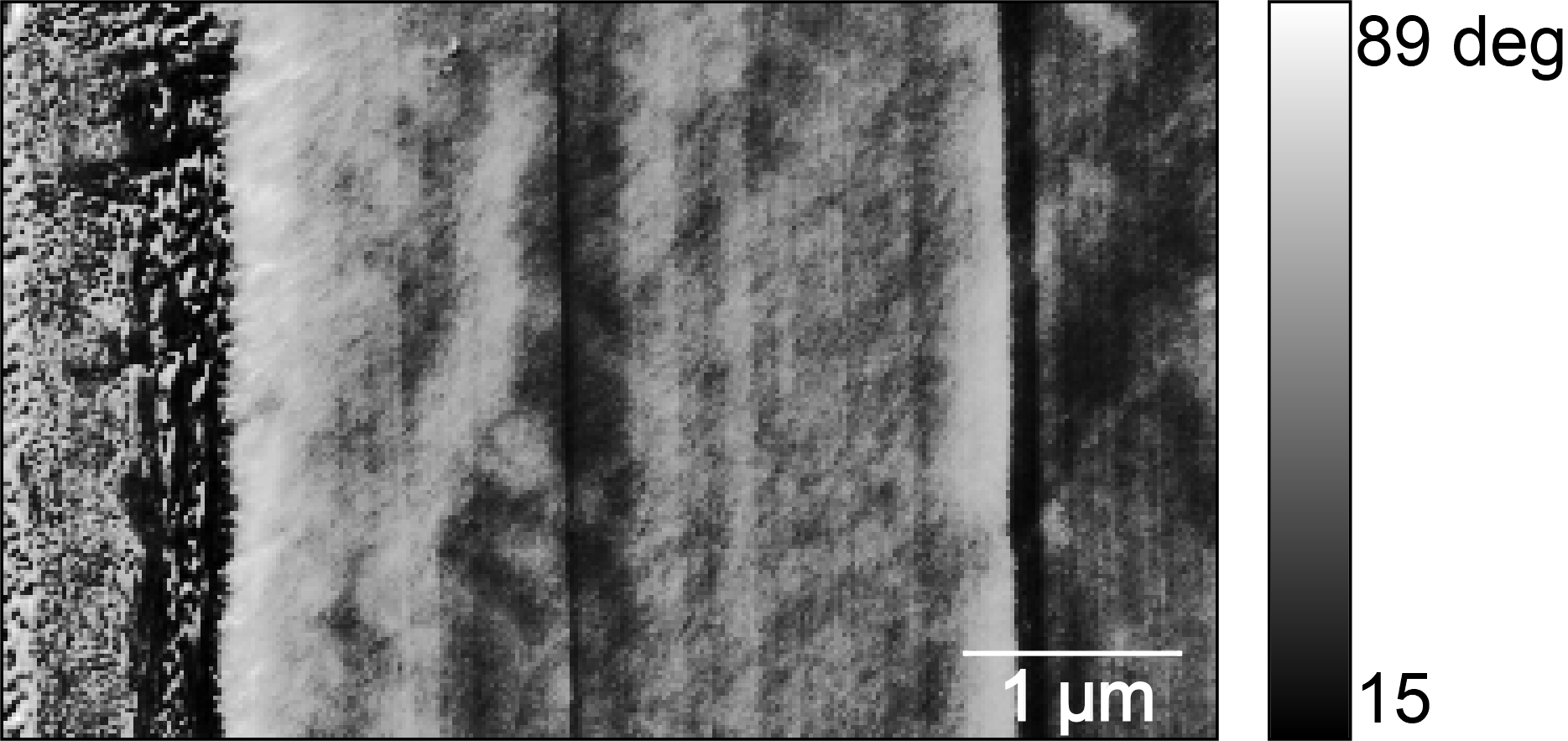


S2

S1

**Fig. S1** AFM phase lag image of the radial-tangential (r̂-t̂) section is shown at a lower magnification than in Fig. 1b. The knife cutting direction indicated by a blue arrow differs from the microfibril orientation indicated by white arrows.

| 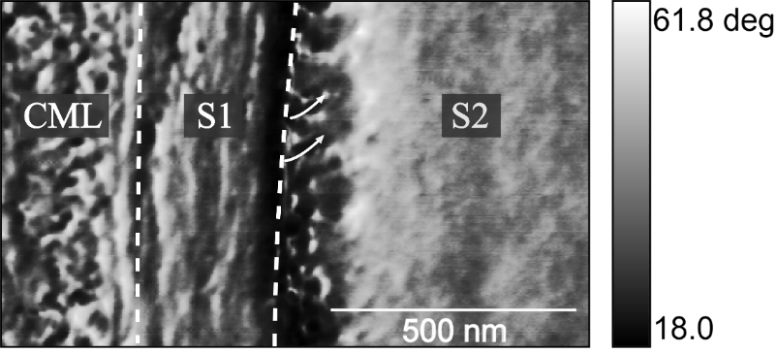  (a) | 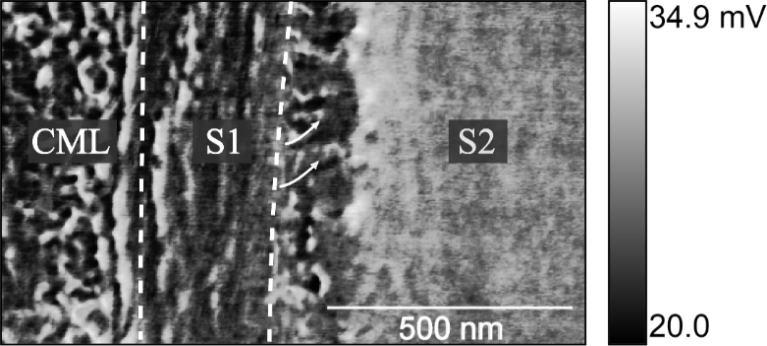  (b) |
| --- | --- |

**Fig. S2** AFM images of the wood cell wall radial-tangential (r̂-t̂) section. **a** phase lag map and **b** dissipation map. The radial component near the S1/S2 interface is indicated with white arrows.

In addition, there is also no evidence that the observed whorl-like microfibril structures are caused by the microtome knife, because the cutting direction is not correlated with the microfibril direction (Fig. S3).


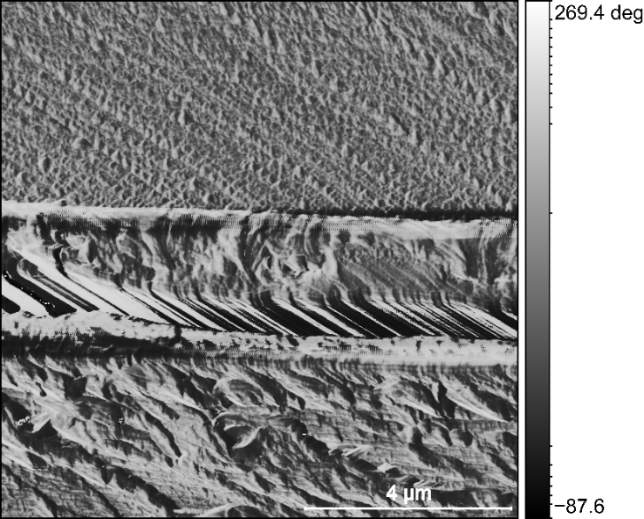


**Fig. S3** AFM phase lag image of a radial-longitudinal (r̂-l̂) section containing the whorl-like structure (black box) shown in Fig. 2c. The knife cutting direction is indicated by a blue arrow.

TEM is an established method for imaging the microfibril structure of cell walls (e.g. Reza et al. 2015, Toumpanaki et al. 2020). However, it requires ultrathin samples (< 100 nm), and both the thinning methods and the TEM imaging can introduce damage to the cell wall material. In this study, samples are thinned by the widely used method of ultramicrotomy as well as by focused ion beam (FIB) machining. The FIB preparation with 30 keV Ga^+^ ions leads to irradiation damage, including the destruction of the microfibril structure and sputtering, as well as to Ga contamination. High currents and doses lead to characteristic dark spots in the TEM image, which can be avoided by working with lower doses. However, other damage produced by the Ga ion beam cannot be completely ruled out. Ultramicrotomy also produces damage. The embedding material required for mechanical stability during microtome slicing, e.g., epoxy resin, is assumed to be only located in the lumen of the cells and not in the cell walls, but some studies reveal contradicting results (Coste et al. 2021). Hence, it cannot be entirely ruled out that embedding affects the microfibril structure. Further, the microtome cutting will deform the surface region, possibly leading to surface features. There is some hope to distinguish the cutting-induced surface features since they typically show a dependence on the cutting direction.

Despite all of the concerns about possible FIB and ultramicrotome damage, the TEM images of lamella prepared by the two methods show the same microfibril arrangements (Fig. 2 b and S4). This is a strong indication that the preparation methods do not significantly change the microfibril structure.


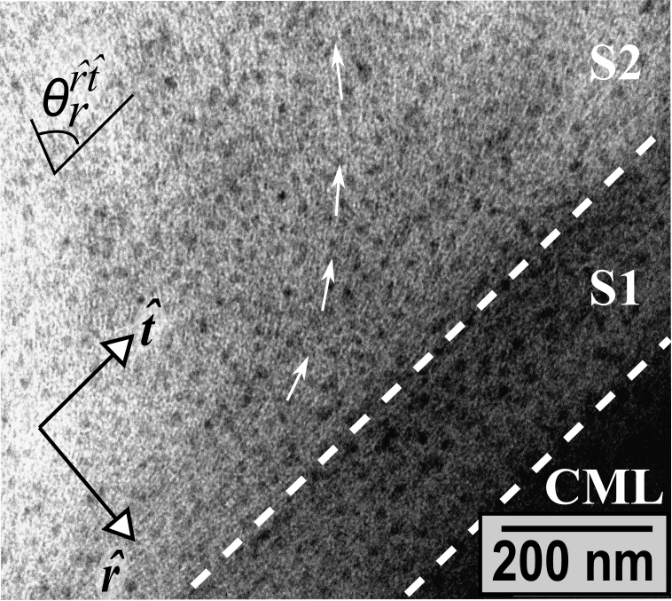


**Fig. S4** TEM image of a FIB prepared radial-tangential (r̂-t̂) section (reproduced from Maaß et al. (2020) under the terms of the Creative Commons CC-BY license). Microfibril orientation is indicated by white arrows.

**A detailed description of AFM sample preparation and measuring procedure**

The radial-longitudinal (r̂-l̂) and radial-tangential (r̂-t̂) sections of unembedded pine and spruce wood were prepared by first cutting matchsticks from suitable locations and orientations and then shaping their ends into pyramids with a razor blade. The pyramid apex was then sliced off to produce an approximately 1×1 mm^2^ smooth surface, either by removing 10 µm slices from the water-brushed pyramid apex using a Sartorius 31A30 sledge microtome equipped with a disposable steel knife, or using a diamond trim knife in the dry state. Afterward, the samples were air-dried at room temperature for several days.

The moisture content of the specimens was equilibrated in the AFM chamber at 65% RH and 21°C for 12 hours before AFM tests. Imaging of the radial-tangential (r̂-t̂) and radial-longitudinal (r̂-l̂) sections was performed with an Asylum MFP 3D AFM using the bimodal AM-FM viscoelastic mapping method (Proksch and Yablon 2012). This method was selected because it allows higher resolution imaging and is less destructive of the sample surface than contact mode. In the bimodal AM-FM method, two cantilever resonances are excited, controlled, and recorded simultaneously. The lower resonance operates in AM (amplitude modulation) mode, where the amplitude and phase are measured at a fixed frequency and used to determine the loss tangent (tan δ) through the ratio of dissipated to stored power in the AFM tip-sample interaction and to prepare the phase lag (δ) maps. The higher frequency resonance operates in FM (frequency modulation) mode, where the drive amplitude and resonance frequency are recorded at fixed cantilever amplitude and fixed phase lag of δ=90°. The resonance frequency is a measure of the sample stiffness (modulus map), and the amplitude contains information about dissipation (dissipation map).

**A detailed description of TEM sample preparation**

To prepare TEM lamella by FIB, cubes (0.5 cm × 1 cm × 1.5 mm) of unembedded pine wood were sectioned by microtome in 60-100 µm thick tangential and cross-sectional slices. The slices were then cut into mm-size pieces with a razor blade. The pieces were stained with a 1 % KMnO_4_ aqueous solution for 30 min, then washed with distilled water and dried on a heating plate at 100°C for a few minutes. Afterwards, the slices were glued with silver paint on a semicircular TEM grid. For preparation of the radial-longitudinal (r̂-l̂) sections, the longitudinal axis of the slice was fixed perpendicular to the straight edge of the semicircle. Then, the slices were coated with a few nanometers of amorphous carbon to avoid beam charging problems in the FIB. Finally, a lamella (15 µm x 15µm x 100 nm) was prepared by standard FIB milling.

The TEM lamella prepared by ultramicrotome were sliced as 100 nm-thin cross-sections from epoxy embedded pine wood cubes (0.5 cm × 0.5 cm × 1.5 mm) with a diamond knife and collected on TEM grids. The sections were post-stained with one drop of a 1 % KMnO_4_ aqueous solution.
